# Inflammatory Microenvironment‐Responsive Hydrogels Enclosed with Quorum Sensing Inhibitor for Treating Post‐Traumatic Osteomyelitis

**DOI:** 10.1002/advs.202307969

**Published:** 2024-03-14

**Authors:** Wenting Zhang, Huidan Lu, Wanying Zhang, Jiahao Hu, Yifei Zeng, Huiqun Hu, Liyun Shi, Jingyan Xia, Feng Xu

**Affiliations:** ^1^ Department of Infectious Diseases The Second Affiliated Hospital Zhejiang University School of Medicine Hangzhou Zhejiang 310009 China; ^2^ Key Laboratory of Multiple Organ Failure (Zhejiang University), Ministry of Education Hangzhou 310053 China; ^3^ Research Center for Life Science and Human Health Binjiang Institute of Zhejiang University Hangzhou 310053 China; ^4^ Department of General Surgery Sir Run‐Run Shaw Hospital Zhejiang University School of Medicine Hangzhou Zhejiang 310016 China; ^5^ Institute of Translational Medicine Zhejiang Shuren University Hangzhou Zhejiang 310015 China; ^6^ Department of Radiation Therapy The Second Affiliated Hospital Zhejiang University School of Medicine Hangzhou Zhejiang 310009 China

**Keywords:** bacterial plasmolysis, drug‐resistant bacteria, post traumatic osteomyelitis, quorum sensing inhibitor, responsive biomaterial

## Abstract

Non‐antibiotic strategies are desperately needed to treat post‐traumatic osteomyelitis (PTO) due to the emergence of superbugs, complex inflammatory microenvironments, and greatly enriched biofilms. Previously, growing evidence indicated that quorum sensing (QS), a chemical communication signal among bacterial cells, can accelerate resistance under evolutionary pressure. This study aims to develop a medical dressing to treat PTO by inhibiting QS and regulating the inflammatory microenvironment, which includes severe oxidative stress and acid abscesses, through a reactive oxygen species (ROS)‐responsive bond between N1‐ (4‐borobenzoyl)‐N3‐(4‐borobenzoyl)‐the N1, the N1, N3, N3‐tetramethylpropane‐1,3‐diamine (TSPBA) and polyvinyl alcohol (PVA), and the amino side chain of hyperbranched polylysine (HBPL). Physically enclosed QS inhibitors subsequently exerted the antibacterial effects. This hydrogel can scavenge hydrogen peroxide (H_2_O_2_), superoxide anion free radical (·O_2_^−^), hydroxyl radicals (·OH) and 2,2‐di(4‐tert‐octylphenyl)‐1‐picryl‐hydrazyl (DPPH) to reduce oxidative stress and inhibit “bacteria‐to‐bacteria communication”, thus clearing planktonic bacteria and biofilms, accelerating bacterial plasmolysis, reducing bacterial virulence and interfering with membrane transport. After in vivo treatment with hydrogel, nearly all bacteria are eliminated, inflammation is effectively inhibited, and osteogenesis and bone repair are promoted to facilitate recovery from PTO. The work demonstrates the clinical translational potential of the hydrogel in the treatment of drug‐resistant bacteria induced PTO.

## Graphical abstract

Inflammatory microenvironment targeted hydrogel promotes osteogenesis and inhibits inflammation in post‐traumatic osteomyelitis by responding to severe oxidative stress or acid abscess environment, and subsequently releasing quorum sensing inhibitors. It can inhibit “bacteria‐to‐bacteria communication” to clear planktonic bacteria and biofilms, reduce bacterial virulence, accelerate bacterial plasmolysis and interfere with bacterial mambrane transport, while improving the inflammatory microenvironment.

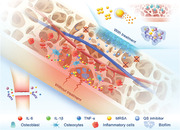

## 1 Introduction

As one of the most colossal challenges in clinical practice, osteomyelitis has imposed huge health‐care and economic burdens.^[^ ^1^ ^]^ The estimated incidence of osteomyelitis has been steadily rising,^[^ ^2^ ^]^ especially post‐traumatic osteomyelitis (PTO), which usually occurs secondary to severe open fractures of long bones, bone surgery and graft infection.^[^ ^3^ ^]^

Currently, surgery and antibiotics are the leading treatments available for PTO.^[^ ^4^ ^]^ However, conventional therapeutic regimens remain unsatisfactory.^[^ ^5^ ^]^ With poor blood flow in PTO, bacteria can adhere to the surface of tissues in planktonic forms and then be encapsulated in their own secreted mucilaginous matrix to form biofilms.^[^ ^6^ ^]^ At this time, dead bone easily impedes the penetration of drugs. Therefore, a high dose of antibiotics is needed to reach the minimal antibacterial concentration in the lesion, which usually leads to multiple side effects and even the evolution of drug resistance. Methicillin‐resistant *Staphylococcus aureus* (MRSA) is the most prevalent superbug during PTO treatment.^[^ ^7^ ^]^ MRSA tends to form acidic abscesses, leaving the bone marrow cavity in a microenvironment with a pH of approximately 5.5–6.5. Here, the massive aggregation of neutrophils forms a barrier that prevents host immune cells from killing bacteria.^[^ ^8^ ^]^

In general, biofilm formation and the genome evolution of drug resistance may be associated with the quorum sensing (QS) and metabolic system,^[^ ^9^ ^]^ through which group behaviors and metabolism are coordinated through intercellular chemical communication.^[^ ^10^ ^]^ In the process of survival and reproduction, bacteria have evolved this unique strategy to adapt to the surrounding environment.^[^ ^11^ ^]^ When the microbial population densities increase, bacterial cells excrete or secrete chemical signals into the environment. At sufficient concentrations, signaling molecules alter the expression of specific genes through signal transduction to regulate the characteristics of microbial populations.^[^ ^12^ ^]^ Bacteria can better coordinate and survive in a complex environment with “team combat capability” by QS systems for “cell‐to‐cell communication”. Hyperbranched polylysines (HBPL) can effectively destroy biofilms to treat infections by interfering QS systems. As an antibacterial substitute for antibiotics, it performs the function of a cationic polymer and kills planktonic bacteria by electrostatic action.^[^ ^13^ ^]^ Besides, owning both the units of ε‐poly‐L‐lysine (ε‐PL) and α‐poly‐L‐lysine (PLL) enables it to exert antibacterial and cell adhesion promoting effects. However, HBPL is easily deactivated and susceptible to environmental changes, such as hydrolysis, oxidation, photolysis, and proteases activity. Additionally, the clinical translation and therapeutic effects of HBPL are limited by its high toxicity and high costs. Moreover, it is more difficult to achieve an effect in patients with PTO due to the complex anatomy of the skeleton. Therefore, it is crucial to improve the stability and enhance drug utilization.^[^ ^14^ ^]^ Numerous studies have attempted to modify the charge and amino acids of these peptides,^[^ ^15^ ^]^ and build drug delivery and sustained‐release systems, such as various responsive nanomaterials, to maximize their efficacy.^[^ ^16^ ^]^

Recently, the design of intelligent environmentally responsive hydrogels, including temperature‐responsive, pH‐responsive, and photosensitive hydrogels, has received extensive research attention. Hydrogel formation can be triggered by the stimulation of certain responsive molecules. It releases the loaded drugs sustainedly and slowly through solubility differences, physical encapsulation and chemical bonding.^[^ ^17^ ^]^ With specific bacterial characteristics, the inflammatory microenvironment around the PTO lesion is usually characterized by excessive oxidative stress and the accumulation of acidic substances, which provides potential targets for the design of responsive hydrogels. Some studies have been performed on hydrogels to regulate environmental pH^[^ ^18^ ^]^ and scavenge reactive oxygen species (ROS).^[^ ^19^ ^]^ To achieve this function, chemically sensitive bonds are typically designed in hydrogels. Respondent carriers for ROS include oxalate polymers, selenium polymers, and sulfur polymers, while respondent carriers for pH include hydrazone and arylhydrazone bonds. The ROS‐responsive effects of phenylboronic ester linkages have been thoroughly studied in the fields of malignancies and cardiovascular disorders.^[^ ^20^ ^]^ In antibacterial studies, hydrogels based on phenylboronic ester linkages have shown superior antibacterial and antioxidant properties in chronic wound healing.^[^ ^21^ ^]^ In past studies, phenylboronic acid‐modified hyaluronic acid and polyvinyl alcohol (PVA) have been successfully synthesized as injectable hydrogels.^[^ ^22^ ^]^ In addition, the side chain of HBPL contains a large number of amino groups, which results in positive pH acid‐responsive effects.^[^ ^23^ ^]^

In this study, we designed an intelligent responsive hydrogel that targets severe oxidative stress and acidic abscesses for the inflammatory microenvironment, choosing PVA as the cornerstone and cross‐linking ROS‐sensitive substances. The hydrogel physically encapsulated nonresistant antimicrobial peptides that could kill planktonic bacteria. Additionally, since the hydrogel inhibited the QS system, it helped destroy the biofilms and eliminate the adherent bacterial colonies. Additionally, both the units of ε‐PL and PLL also enabled HBPL to promote cell attachment, proliferation, and differentiation. Excellent antimicrobial and bone‐repairing effects were more easily achieved with the regulated release of drugs. Our work creatively put forward a new way to cut off the pathway of QS to treat refractory PTO caused by drug‐resistant bacteria, which provided a new strategy in removing the biofilms and planktonic drug‐resistant bacteria affecting osteomyelitis healing.

## 2 Results and Discussion

### 2.1 Synthesis and Characterization of Hydrogel

**Figure 1:**
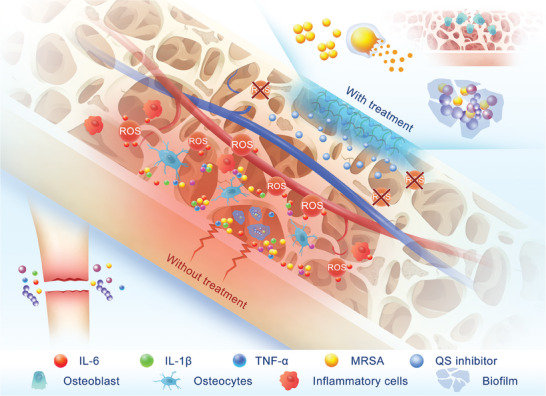
A schematic diagram of HBPL‐gel‐mediated antibacterial activity and therapeutic effect of PTO. Inflammatory microenvironment targeted hydrogel promotes osteogenesis and inhibits inflammation in PTO by responding to severe oxidative stress or acid abscesses environment and releasing QS inhibitors. It can inhibit “bacteria‐to‐bacteria communication” to clear planktonic bacteria and biofilms, reduce bacterial virulence, accelerate bacterial plasmolysis, interfere with bacterial membrane transport, and improve the inflammatory microenvironment.

To target the inflammatory microenvironment while depleting ROS and neutralizing acidic abscesses, we synthesized a ROS and pH dual‐responsive hydrogel carrying the QS inhibitor HBPL. (**Figure** **1**)

**Figure 2:**
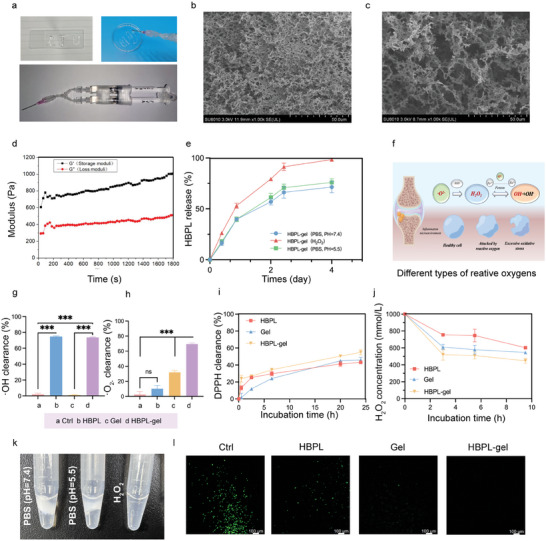
Characterizations of hydrogels. a) Photos of the double‐syringe system and the injectability of the HBPL‐gel. b,c) SEM images of the Gel in PBS solution and H_2_O_2_ solution. d) Storage modulus (G′) and loss modulus (G″) of the Gel at 1hz and 1% dynamic strain. e) Drug release percentage in PBS (pH 7.4), 1 mm H_2_O_2_ and PBS (pH 5.5). f) Different types of ROS. g–j) g)·OH, h)·O_2_^−^, i) DPPH, and j) H_2_O_2_ clearance of hydrogels. k) Photographs of Gel in different solutions. l) Fluorescence microscope images of H_2_O_2_‐stimulated RAW264.7 with different treatments. Data are presented as mean ± SD. (*n* = 3–5 per group, ns: no significance, ^***^ *p* < 0.001).

First, HBPL was synthesized, and the structure was confirmed by mass spectrometry. (Figure S1, Supporting Information) Then, we synthesized N1‐ (4‐borobenzoyl)‐ N3‐ (4‐borobenzoyl)‐ the N1, N3, N3‐ tetramethylpropane (TSPBA) with ROS‐sensitive bonds^[^ ^24^ ^]^ and cross‐linked it with PVA to obtain a stable hydrogel network. The Gel referred to the hydrogel prepared by 5 wt.% TSPBA and 5 wt.% PVA. The HBPL‐gel referred to the Gel encapsulating HBPL. The hydrogels exhibited responsiveness to ROS and pH due to the phenylboronic acid ester bond and the amino groups on the HBPL side chain. Then, the structure of TSPBA was confirmed by nuclear magnetic resonance (Figure S2, Supporting Information) and the structure of the Gel was confirmed by Fourier transform infrared spectrometer (FITR) (Figure S3, Supporting Information). We manufactured the hydrogels in the shape of a double syringe to implement in situ hydrogel formation, as depicted in **Figure** **2**a, and then we demonstrated their injectability by videos (Supplementary File [Link], [Link], Supporting Information). The morphology of the dried HBPL‐gel network was observed by scanning electron microscopy (SEM), with a porous structure (Figure 2b), whereas the micro‐structure became loose after being incubated in hydrogen peroxide (H_2_O_2_) solution for 7 days (Figure 2c). It was obvious that HBPL‐gel swelled in H_2_O_2_ solution to a larger equilibrium volume. Subsequently, we examined the shear thinning properties (Figure S4, Supporting Information). To clarify the mechanical properties of the hydrogels, representative tensile and compressive stress‐strain curves of the hydrogel samples were plotted. The Gel exhibited the best compressive elasticity at 581% pressure strain, with a fracture tensile strength of ≈18.51 kPa (Figure S5, Supporting Information). Correspondingly, the Gel fractured compressively at 63.81% pressure strain (Figure S5, Supporting Information). As a function of time, the shear storage modulus G′ and loss modulus G″ of the hydrogel were monitored. The values of G′ represented the stored energy, while G″ stood for the deformation energy that is lost by internal friction during shearing. As shown in Figure 2d, the shear storage modulus G′ was greater than the loss modulus G″, suggesting that the Gel had superior gelation properties. In addition, we examined the release rate of HBPL in hydrogels (Figure 2e). It was obvious that HBPL in PBS solution without H_2_O_2_ were not totally released and the curve showed a platform. On the contrary, The hydrogel exhibited a faster drug release rate with a higher release peak in acidic and H_2_O_2_ environments. The release of the drugs depended on the concentration difference and the swelling of the Gel. Then we took photos of different types of hydrogels incubated with different solutions (Figure 2k)_._ We found that Gel was not totally degraded in day 7, so the drugs might still remain in the Gel in PBS solution without H_2_O_2_. With the extension of time, the drug inside the Gel diffused outward and the concentration gradient between the Gel and the outside medium gradually decreased, which slowed down the drug release rate to finally reach a platform.

ROS mainly include H_2_O_2_, superoxide anion free radical (·O_2_^−^), hydroxyl radicals (·OH) and superoxide (Figure 2f).^[^ ^25^ ^]^ To evaluate the antioxidant properties of the hydrogel, we examined its ability to scavenge H_2_O_2_, ·O_2_^−^, 2,2‐di (4‐tert‐octylphenyl)‐1‐picryl‐hydrazyl (DPPH), and ·OH (Figure 2g–j). Based on the results, HBPL but not the hydrogel exhibits a considerable scavenging (Figure 2g) ability for ·OH. This might indicate that the amine group of HBPL played a major role in this process. We found that the Gel scavenged the most ·O_2_^−^, while HBPL barely scavenged ·O_2_^−^ (Figure 2h). Within 24 h, the clearance of DPPH by the HBPL‐gel was slightly higher, while the trends were not very different among the other groups. (Figure 2i). Within 10 h, the fastest rate of H_2_O_2_ clearance was achieved by the HBPL‐gel, followed by the hydrogel alone (Figure 2j). Based on the results, we conclude that HBPL contributes to the scavenging of DPPH, ·OH and H_2_O_2_, while the Gel contributes to the scavenging of ·O_2_^−^, ·OH, and H_2_O_2_, which allows the HBPL‐gel to effectively scavenge a variety of ROS. In addition, the capacity of hydrogels to scavenge ROS was investigated by incubating H_2_O_2_‐stimulated RAW264.7 with various hydrogels. The results (Figure 2l) revealed that HBPL, hydrogel, and HBPL‐gel exhibited almost negligible green fluorescence signals, while the control group showed strong green fluorescence of ROS.

In the inflammatory microenvironment, the production of ROS acts as a double‐edged sword. It is generally accepted that ROS can kill bacteria through intense cellular oxidation.^[^ ^26^ ^]^ However, excessive ROS also cause the differential expression of inflammatory genes and damage cellular molecules such as DNA, proteins, and lipids, exacerbate apoptosis of healthy cells and aggravate the inflammatory response, which is not conducive to bone repair.^[^ ^27^ ^]^ In our study, we hypothesized that high ROS produced by bacterial infection leads to increased inflammation, causes oxidative stress damage to surrounding structures and inhibits the growth, repair and healing of bone. Above all, our results demonstrated that the hydrogel performs anti‐inflammatory functions by scavenging ROS. Additionally, the results support that our hydrogels function effectively in an inflammatory microenvironment in ROS scavenging ways.

### 2.2 Antibacterial Study of Drug‐Resistant Bacteria In Vitro

**Figure 3:**
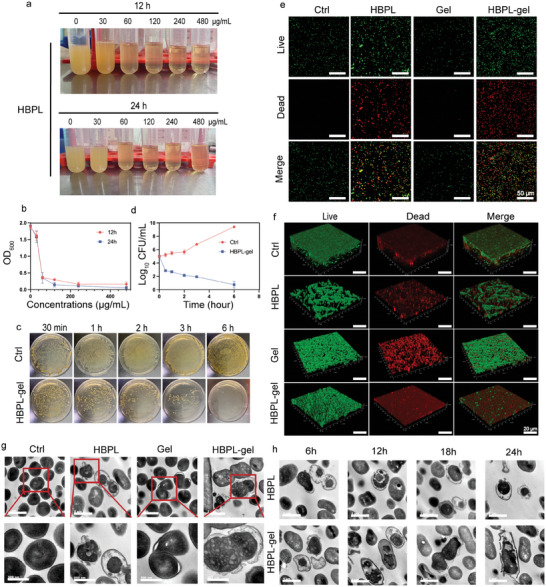
Antibacterial ability to drug‐resistant bacteria of hydrogels in vitro. a) Photographs of MRSA co‐incubated with different concentrations of HBPL. b) OD_600_ changes of MRSA treated by HBPL with different concentrations. c) Agar plate photos of MRSA under different treatments with different time. d) Corresponding CFU changes of MRSA with different time. e) Fluorescence photos of MRSA stained with SYTO 9 (green, live bacteria) and PI (red, dead bacteria) after corresponding treatments. f) 3D confocal laser scanning microscope images of SYTO9/PI‐stained mature MRSA biofilms and different hydrogels treated biofilms. g) Representative TEM images (top) and corresponding higher magnification images (bottom) of MRSA under different treatments. h) TEM images of MRSA treated with HBPL and HBPL‐gel during the process of bacteria killing. Data are presented as mean ± SD. (*n* = 3–5 per group).

Effective antibacterial ability is the most essential property of treatment against PTO caused by drug‐resistant bacteria. First, we took photographs of MRSA incubated with increasing concentrations of HBPL (**Figure** **3**a). As shown, at concentrations higher than 120 µg mL^−1^, the HBPL could eliminate almost all bacteria. Then, we drew concentration‐varying curves of the OD_600_ value of MRSA (Figure 3b). Additionally, the mixtures were coated on trypophan soy agar (TSA) plates at 0.5, 1, 2, 3, and 6 h, and the concentration‐varying curves of colony forming units (CFU) changes were drawn (Figure 3c,d). We found that it could kill almost all bacteria within 6 h. Subsequently, we detected the live (green) and dead (red) states of bacteria by a SYTO9/PI staining kit (Figure 3e). The results indicated that the HBPL‐gel and HBPL groups exhibited better antibacterial effects than that of the other groups. Live (green) and dead (red) fluorescence staining of biofilms (Figure 3f) also demonstrated that our hydrogels disrupted the formation of mature bacterial biofilms. Transmission electron microscope (TEM) was performed to explore the potential mechanism and further study the changes in the internal morphology of bacteria under different treatments (Figure 3g,h). Untreated bacteria showed continuous and intact cell membranes with normal morphology. However, MRSA treated with HBPL‐gel showed abnormal morphology, including cell wall rupture, cell membrane shrinkage, loss of cell contents, and even “plasmolysis” similar to plant cells. However, the attack of simple hydrogel on bacteria was not obvious when some bacteria adhered to each other. As time went by, the antibacterial effect was more obvious. However, the effects of HBPL‐gel and HBPL were similar, which indicated the main role of HBPL cation. The encapsulation of Gel had no effect on the bacterial ability of HBPL. These results indicated that our hydrogels might kill planktonic bacteria and mature biofilms by attacking the cell wall or cell membrane or changing the internal and external osmotic pressure.

### 2.3 Regulation of MRSA Transcriptome by HBPL‐Gel

**Figure 4:**
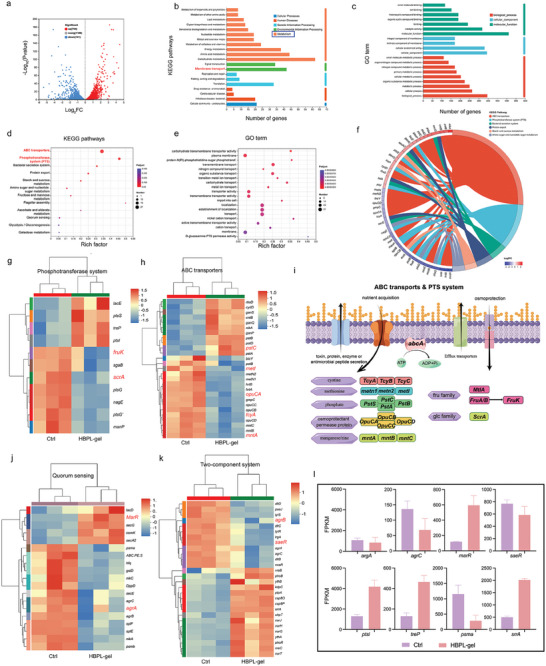
Transcriptome sequencing analysis of MRSA treated with HBPL‐gel. a) Volcano plot analyses of total differentially expressed genes (DEGs) between the HBPL‐gel treatment group and the PBS group. Blue and red dots represent down‐regulated and up‐regulated genes. Grey dots represent the genes not statistically different. b) KEGG analysis of DEGs. c) GO analysis of DEGs. d) Bubble plot of KEGG analyses in the “Membrane transport” pathway. e) Bubble plot of GO term in the “Membrane transport” pathway. f) KEGG chord diagram in the “ABC transporters” and “phosphotransferase system” pathway. g) Clustering heat map of genes associated with phosphotransferase system. h) Clustering heat map of genes associated with ABC transporters. i) Schematic representation of the ABC transports and PTS system. j) Clustering heat map of genes associated with quorum sensing. k) Clustering heat map of genes associated with two‐component system. l) Fragments Per Kilobase of exon model per Million mapped fragments (FPKM) of some important genes.

To explore the mechanism underlying HBPL‐gel activity in bacterial treatment, we analyzed the transcriptome sequencing (RNA‐seq) results of MRSA with or without treatment. In the volcano plot of differences between groups (**Figure** **4**a), upregulated and downregulated genes are shown in red and blue, respectively. To further clarify the effect of HBPL‐gel on the biological function and gene pathway of MRSA, we conducted gene ontology (GO) and Kyoto Encyclopedia of Genes and Genomes (KEGG) analysis (Figure 4b,c). It was found that the functional differences between the control and treatment groups were mainly focused on the “Metabolism” pathway. And “Membrane transport” pathway also played an important role between two groups. Subsequently, as shown in Figure 4d,e, we performed KEGG and GO enrichment analysis in “Membrane transport” pathways and found that the most significant differences among them were in “ATP‐binding cassette (ABC) transporter” and “phosphotransferase system (PTS)”. Subsequently, we performed a chord diagram of KEGG analysis in “ABC transporter” and “PTS system” pathways (Figure 4f). Then we performed a clustering analysis of ABC transporter‐related genes, and the heatmap (Figure 4h) showed that the genes were upregulated and downregulated among them. Among them, some small molecule substances, such as nickel, iron, phosphate, and spermidine transporters, were upregulated in the treatment group, such as the nickel transporter‐related gene *nikA* and phosphate transporter‐related gene *pstC*. The manganese/zinc‐regulating genes *mntA, methionine* transporter‐related gene *metl* and oligopeptide transporter‐related gene *opuCA* were downregulated. Therefore, we hypothesized that bacterial membrane transport function was more disorganized after treatment, and it was difficult to maintain normal osmotic pressure and cell membrane homeostasis. In contrast, PTS regulates the transport of one particular substance, that is inorganic phosphate.^[^ ^28^ ^]^ Likewise, we plotted the differentially expressed PTS‐related differential genes in a clustering heatmap (Figure 4g). According to the results, we generated a schematic diagram of some important genes (Figure 4i). Besides, genes associated with QS are plotted as heatmaps (Figure 4j), indicating a substantial reduction in their expression. In MRSA, accessory gene regulator (*agr*) was thought as the main mechanism in QS systems to modulate bacterial cell density and regulate biofilm production.^[^ ^29^ ^]^ Both *agrA* and *agrC* (Figure 4l) genes were significantly down‐regulated in HBPL‐gel treated MRSA. Multiantibiotic resistance regulators *marR* functioned as a repressor of genes encoding efflux pumps.^[^ ^30^ ^]^ It could inhibit the expression of downstream drug resistance genes and was up‐regulated in HBPL‐gel treated MRSA (Figure 4l). Therefore, the QS system was inhibited and the ability of adaptive evolution to resist the biotics was weakened under treatment. Metabolic and virulent properties of bacteria were related to two component systems. We plotted a heatmap with two‐component system‐related genes (Figure 4k). It revealed that a majority of pathogenesis‐related toxin genes were downregulated. The pro‐inflammatory cytokine response induced by *SaeR* in MRSA infection was downregulated in HBPL‐gel group^[^ ^31^ ^]^ (Figure 4l).

The QS system, as a signal for bacteria to communicate with each other, can promote biofilm formation to a large extent. HBPL is a cationic antimicrobial peptide that contains many amino groups in the side chains. Similar to the antibacterial mechanism of chitosan in general,^[^ ^32^ ^]^ we speculate that this positively charged macromolecule can cover the bacterial surface and form a dense membrane, blocking the exchange of nutrients or metabolic waste on the surface of the bacteria and inhibiting bacterial growth. This electrostatic effect can also change the membrane wall permeability and damage the membrane wall structure, causing internal osmotic pressure imbalance, ion and water molecule outflow, and internal pressure drop, leading to bacterial death. In addition, amino acids can chelate with metal ions needed to maintain cell membrane stability and normal cell metabolism, interfering with normal bacterial metabolism, and inhibiting bacterial growth.

According to our results, ABC transporters played a significant role in the process by which bacteria are killed. The ABC transporter was first discovered in bacterial nutrient uptake studies during the 1970s.^[^ ^33^ ^]^ It is a kind of bacterial plasma membrane enzyme that transports ATPase. The transporters can transport many different substrates, including various minerals and organic ions, oligosaccharides, lipids, and small proteins.^[^ ^34^ ^]^ In bacteria, ABC transporters are mainly involved in nutrient uptake, metabolic pathways,^[^ ^35^ ^]^ excretion of toxic compounds, and biofilm formation, which are closely related to bacterial drug resistance.^[^ ^36^ ^]^ Bacteria living in a hyperosmotic environment have developed a complex osmoregulatory system over a long period of evolution to counteract external osmotic stress. The sugars, amino acids and alcohols in the bacterial cytoplasm can be used as osmoprotectors to increase the osmotic pressure of the cell and maintain the stability of the cell membrane.^[^ ^37^ ^]^ Therefore, membrane transport is important for the survival of bacteria.

After the problem involving mutual adhesion between bacterial populations was solved, the morphology of individual bacteria also appeared significantly abnormal, as shown by our TEM images (Figure 3g,h). In the treated bacteria, the cell membranes and cell walls were ruptured and damaged, bacterial content had escaped, swelling was evident, and a “plasmolysis” phenomenon was observed similar to that of plant cells. In previous studies, TEM was used to observe the ultrastructural changes of hemolytic grape bulb cells exposed to polycationic chitosan. Part of the cell membrane detached from the cell wall at the interaction site, resulting in a “vacuole‐like” structure, which allowed ions and water molecules to escape. This structural change cause decreased internal pressure and led to bacterial death. Therefore, we explored the genes related to the cell membrane. Our RNA‐seq analysis results are consistent with previous TEM results. In addition to inhibiting the bacterial QS system, the treatment group could disrupt the bacterial cell membrane transport system, affect the normal osmotic pressure of bacteria, and interfere with bacterial metabolism. As a result, the bacterial cell membrane and cell wall ruptured, the cell body became swollen, and plasmolysis occurred.

### 2.4 Healing of PTO In Vivo

**Figure 5:**
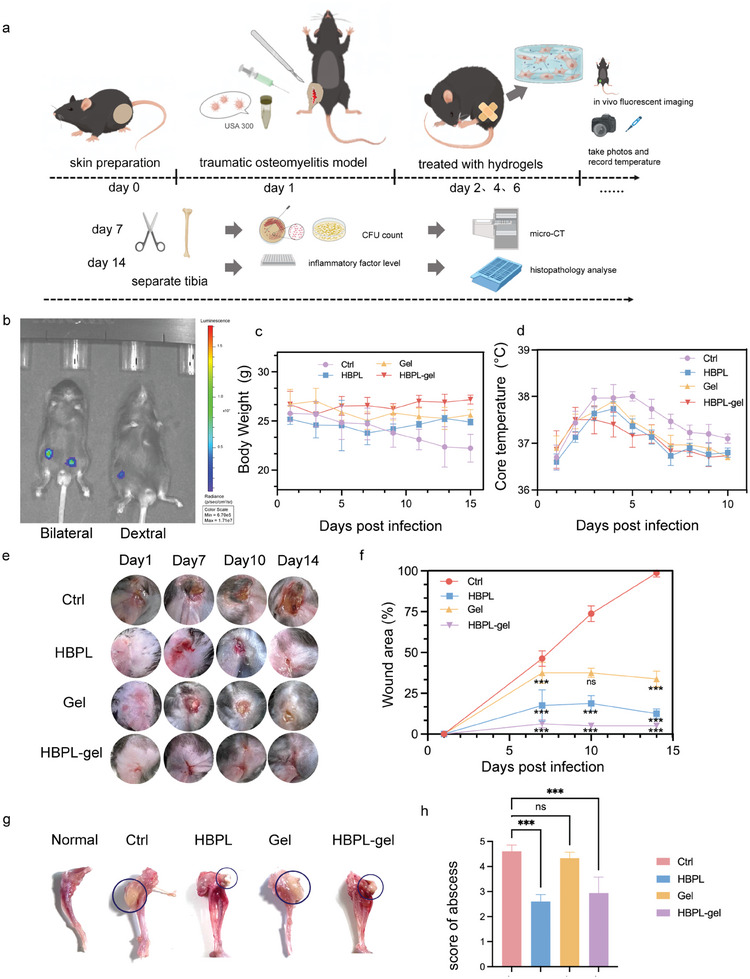
Healing of PTO in vivo. a) Summary diagrams of PTO model schematic, treatment strategies and detection methods. b) Luminescence images of unilateral and bilateral infected mice. c) Body weight changes of PTO mice infected with MRSA in corresponding groups from day 1 to day 15. d) Core body temperature of PTO mice. e) Representative photos of wounds in operative sites with different treatments on day 1,7,10, and 14. f) Wound area changes from day 1 to day 14 and wound size on day 14 of the control group was used as 100% benchmark. g,h) Representative photos and scores of abscess degree of tibia with different treatments. Black circle: abscess around the tibia. Data are presented as mean ± SD. (*n* = 3‐5 per group, ns: no significance, ^***^ *p* < 0.001)

Given the promising therapeutic potential of the hydrogel in vitro, we further evaluated its impact on the treatment of PTO in vivo. To estimate the antibacterial ability to eliminate MRSA in vivo, we established a mouse PTO model infected with MRSA (**Figure** **5**a). To verify the success of model building, we photographed unilateral and bilateral infected mice with an In Vivo Imaging System (IVIS) (Figure 5b). Then, the mice were randomly divided into 4 groups and treated with PBS, HBPL, hydrogel, and HBPL‐gel, with scheduled drug treatment. We observed the general growth of these mice and found that the control infected group had the most difficulty moving their right foot, followed by the hydrogel group. Except for the control group, in which the mean body weight decreased continuously (Figure 5c), the body weights of the mice in the remaining groups did not show significant fluctuations. By measuring the mean anal temperature of the mice over time (Figure 5d), we found that the body temperature of the mice peaked on days 3–5 after infection and then decreased. In the treated group, the trend observed for the temperature changes seemed more moderate. Figure 5e demonstrated the healing process at the surgical site surface after different treatments over time. The skin at the surgical site in the control group could not heal naturally. In contrast, the surgical site wound area was over 50% smaller in the hydrogel group than the control group (Figure 5f). The hydrogel dressing might have controlled the extent of wound expansion by closing the wound; thus, further invasion of foreign bacteria could be prevented, or the inflammatory response induced by ROS stress could be reduced. The extent of trauma was not increased after 7 days in the HBPL and HBPL‐gel groups, and >90% of the trauma was repaired and healed on day 14. Therefore, we inferred that these two groups played a specific role in traumatic bone infection. MRSA could infect bone marrow and soft tissue surrounding by forming robust staphylococcal abscess communities (SACs) during osteomyelitis. The SACs were commonly used for diagnosing and classifying the stage of osteomyelitis as SACs could greatly increase the severity.^[^ ^38^ ^]^ We isolated the tibia in each group separately. The soft tissues around the bone in the control groups showed obvious abscesses compared to the treatment groups (Figure 5g). We scored the abscesses according to their size (Figure 5h) and found a significant decrease in the extent of abscesses in the HBPL and HBPL‐gel groups. The antibacterial effect inhibited the generation of abscesses after bone infection. This might be due to the pH‐responsive bond and the large number of amino side chains of HBPL that neutralize the acidic abscess environment.

### 2.5 Bactericidal Effect of the Hydrogel In Vivo

**Figure 6:**
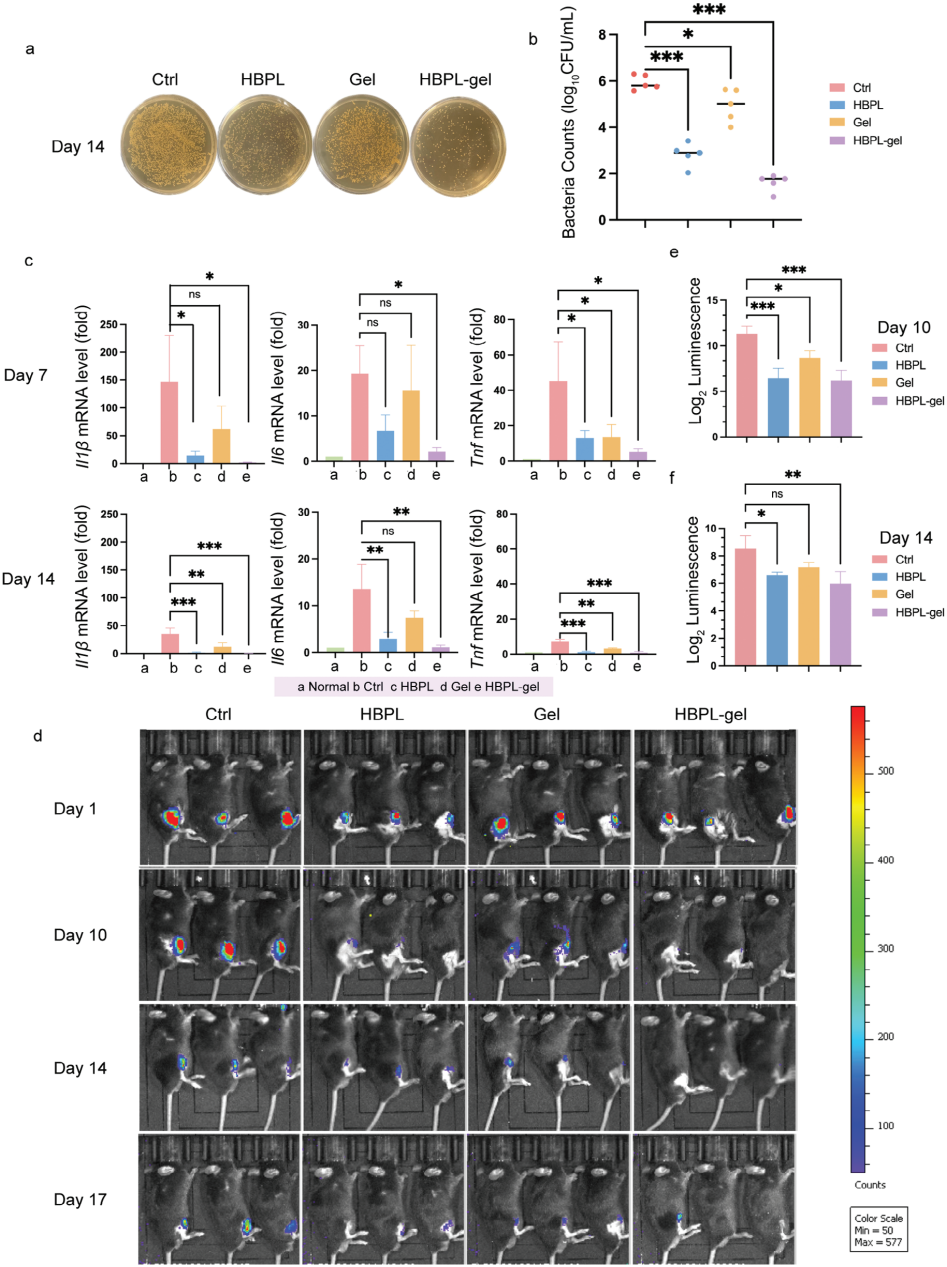
Antibacterial effects of hydrogels in vivo. a,b) Agar plate photographs and quantitative analysis of MRSA under different treatment on day 14. c) mRNA expression level of *Il‐1β*, *Il‐6*, and *Tnf* under different treatments on day 7 and day 14. d–f) Bacterial luminescence images and chemiluminescence quantitative analysis under different treatments. Data are presented as mean ± SD. (*n* = 3–5 per group, ns: no significance, ^*^ *p* < 0.05, ^**^ *p* < 0.01, ^***^ *p* < 0.001).

To investigate the antibacterial activity of hydrogels in vivo, we excised the tibia and surrounding soft tissues on day 14, and ground and cultured the isolated bacteria on TSA plates. By day 14, the bacterial count was significantly reduced in all other groups compared to the control group, and the HBPL‐gel group experienced the largest drop (**Figure** **6**a). After counting, we discovered that the bacterial burden in the infected group was significantly higher than that in the other treatment groups (Figure 6b).

Notably, the inflammatory microenvironment around the fracture area accumulated an abundance of proinflammatory cytokines.^[^ ^39^ ^]^ Through quantitative real‐time PCR (qPCR), we noticed that the expression levels of *Il‐1β*, *Il‐6* and *Tnf* were higher in infected tissue than in healthy tissue (Figure 6c). Although the trends observed for changes in of pro‐inflammatory cytokine were comparable in all groups on days 7 and 14 post infection, the control group displayed the highest levels, the hydrogel group exhibited the second‐highest levels, and the HBPL‐gel group displayed the lowest levels. Pro‐inflammatory cytokines were modulator of host resistance to bacterial infection.^[^ ^40^ ^]^ The low pro‐inflammatory cytokines were consist with the potent anti‐bacterial effects of HBPL. This illustrates that the treatment group, particularly the HBPL‐gel group, can effectively reduce local inflammation levels in vivo. Compared to day 7, *Il‐1β*, *Il‐6*, and *Tnf* were lower in all groups on day 14. As a result of self‐regulation in mice, the degree of inflammation surrounding the fracture wound decreases during the natural progression of PTO. Additionally, the suppression of inflammation promotes wound healing and the repair of nearby bone and connective tissues. Besides, the swelling ability of hydrogel allowed it to absorb inflammatory exudate surrounding wound and keep moist, which could also adhere closely to the wound and reduce the contact of bacteria. The ROS‐scavenging ability of the responsive hydrogel also offered a certain effect on reducing inflammation. Therefore, we concluded that our antimicrobial hydrogel could regulate the local inflammatory microenvironment.

To effectively localize the site of bacteria, validate the successful establishment of the PTO model, and monitor the changes in bacterial load in the bone marrow cavity in real time, we photographed each group of mice after infection with MRSA expressing luciferase (Luci‐MRSA) by an IVIS. Based on the chemiluminescence signal (Figure 6d) recorded by the imaging system, it could be confirmed that the HBPL‐gel could significantly reduce the bacterial load at the fracture site in mice with PTO and effectively kill MRSA in vivo. On day 10, significant differences in bacterial load were observed in all groups after treatment compared to the control group, while on day 14, the HBPL‐gel group still exerted a significant effect (Figure 6e,f). In addition, we found that the chemiluminescence levels of mice in each group showed a decreasing trend over time. This is similar to the trend observed for pro‐inflammatory cytokines, which may function as a recovery mechanism of the mice.

### 2.6 Anti‐Inflammatory Effect of the Hydrogel In Vivo

**Figure 7:**
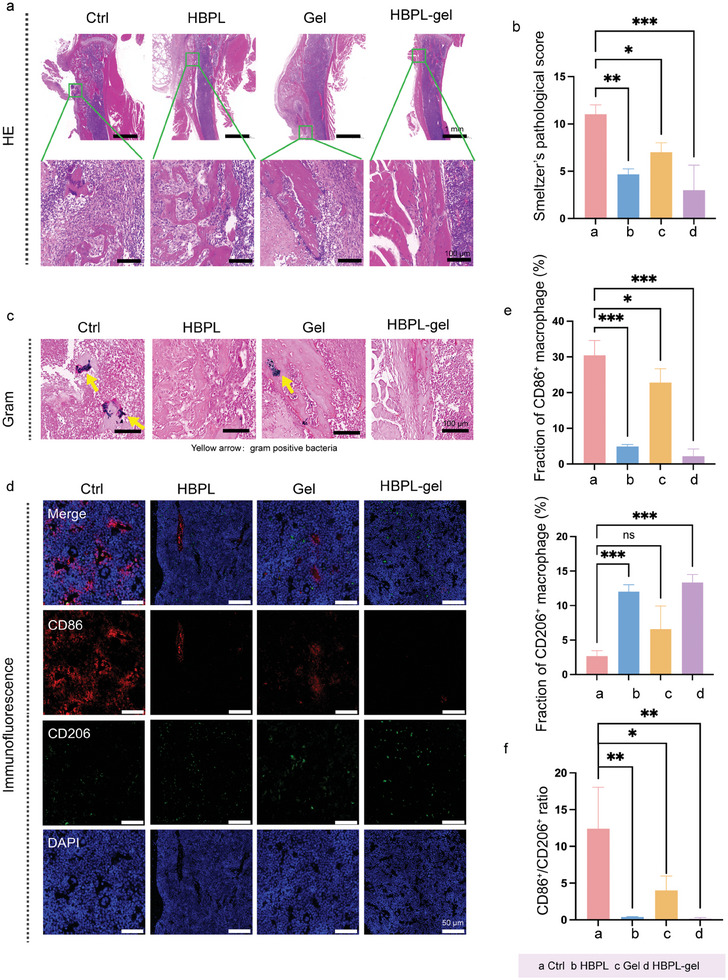
Anti‐inflammatory effects of hydrogels in vivo. a) H&E staining of inflammatory cell infiltration in tibia under different treatments. b) Smeltzer's pathological score. c) Gram staining in tibia under different treatments. (Purple plots: gram‐positive bacteria. Yellow arrow: bacterial colony). d) Immunofluorescence staining of DAPI for cell nucleus (blue), CD86^+^ for identifying pro‐inflammatory phenotype macrophages (red) and CD206^+^ for identifying anti‐inflammatory phenotype macrophages (green) under different treatments. e) Fraction of CD86^+^ and CD206^+^ macrophages. f) CD86^+^/CD206^+^ macrophages ratio. Data are presented as mean ± SD. (*n* = 3–5 per group, ns: no significance, ^*^ *p* < 0.05, ^**^ *p* < 0. 01, ^***^ *p* < 0.001).

After implementing the mouse PTO model and applying different treatments, we used ROS Brite 700 to detect ROS scavenging ability. The results of in vivo imaging (Figure S6, Supporting Information) showed that the cells in the infected area experienced a severe oxidative stress. The ROS level in all treatment groups were lower than that in control group, which might contribute to the ROS scavenging ability of the Gel and the antibacterial ability of the HBPL. In addition, hematoxylin and eosin (H&E) staining (**Figure** **7**a) was performed to further evaluate the healing conditions of PTO. It was obvious that MRSA infected bone showed severe cortex destruction without healing tendency, and a large number of inflammatory cells were infiltrated in the surrounding soft tissue. In contrast, bone showed relatively continuous cortical bone in HBPL and Gel groups, and the proliferation and expansion of bone indicated the healing tendency. It exhibited the most excellent healing properties in HBPL‐gel groups. Subsequently, we conducted Smeltzer pathology scoring independently by two members and pooled the scores (Figure 7b).^[^ ^41^ ^]^ The results now provide evidence for the anti‐inflammatory effect of our antibacterial hydrogels.

Subsequently, we harvested the tibias. Then, gram stains were performed to identify the fracture regions infected with MRSA. Here, gram‐positive bacteria were represented in purple plots, and yellow arrows denoted the locations of bacterial clusters (Figure 7c). The results were consistent with observations in previous studies showing in which infected mice exhibited visible bacterial clusters in the fracture regions, while no obvious bacterial clusters were observed in the HBPL‐gel group. Overall, we verified that HBPL‐gel can function as a powerful bactericidal agent in animals.

Macrophages are among the most vital immune cells involved in inflammation. Previous studies have demonstrated that the inflammatory response after acute bone injury hampers bone repair.^[^ ^42^ ^]^ Inflammatory repair is negatively impacted by inflammatory cytokines, which are mainly released by proinflammatory macrophages.^[^ ^43^ ^]^ Anti‐inflammatory macrophages, on the other hand, exhibit anti‐inflammatory properties and contribute to bone repair. The conversion of proinflammatory macrophages into anti‐inflammatory macrophages promotes tissue healing.^[^ ^44^ ^]^ Based on this, we analyzed macrophages (Figure 7d) in the tibia by immunofluorescence. According to the data (Figure 7e,f), the percentage of CD86^+^/CD206^+^ macrophages were significantly lower in the HBPL‐gel group than the control group. A similar trend was observed for the HBPL group. As bacteria were eliminated early by HBPL and the ROS were cleared by the Gel, these treated groups showed lower bacterial load and milder inflammatory response, so they had lower CD86+/CD206+ ratio. Consequently, we inferred that the application of hydrogels would enhance the anti‐inflammatory effect and facilitate bone healing.

### 2.7 The ability of the Hydrogel to Promote Osteogenesis In Vivo

**Figure 8:**
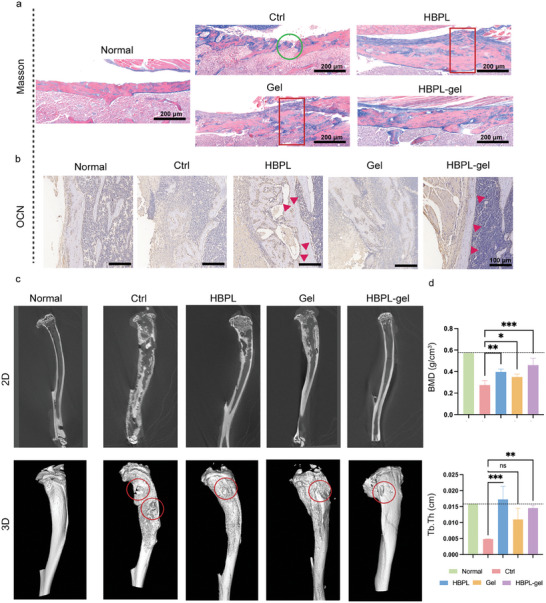
The ability to promote osteogenesis of hydrogel in vivo. a) Masson staining in tibia under different treatments. Green circle: the rupture of the tibia. Red box: swollen part of the tibia. b) Micrographs of OCN in immunohistochemistry slices. Red triangle: OCN. c) Representative 2D and 3D micro‐CT images under different treatments. d) Analysis of BMD and Tb.Th on day 14. Data are presented as mean ± SD. (*n* = 3–5 per group, ns: no significance, ^*^ *p* < 0.05, ^**^ *p* < 0.01, ^***^ *p* < 0.001).

To investigate the therapeutic effect of hydrogel on bone defects and osteogenic repair in vivo, we first evaluated its histopathological changes by Masson staining. **Figure** **8**a presents the fibrous histomorphology of the proximal tibia and cortical bone. In normal tibia, it showed a continuous cortex, dense red‐stained lamellar bone. When infected by MRSA, it was obvious that the bone cortex experienced a severe rupture (green circle) and disordered bone structure, which indicated that there was no significant healing occurred. Compared to the untreated mice, animals in HBPL and Gel groups showed more continuous bone tissues that accompanied by the new bone formation (blue staining) which indicated the growth and preliminary repair of the bone. However, it still showed the swollen part (red box) in these two parts. HBPL group showed better bone continuity and less new bone formation indicating the later period of bone repair. The HBPL‐gel group showed best bone repair as its almost recovered bone continuity replaced by lamellar bone covered by periosteum, and the absorption of excess bone tissue. In summary, our antibacterial hydrogels can also promote the formation of new bone. HBPL might exert an antibacterial effect while the Gel exerted the anti‐inflammatory effect. Subsequently, the expression of osteocalcin (OCN) in the tibia was detected by immunohistochemistry. It is an ossification marker that is a hormone‐like polypeptide generated and emitted by osteoblasts. Figure 8b demonstrates how the hydrogel treatment improved OCN expression in the tibia, which suggested that antibacterial therapy also promoted osteogenesis concurrently.

We performed micro‐CT on day 14 after treatments to evaluate the bone morphology, and the direct outcome is reported in Figure 8c. The morphology of the uninfected tibia was observed to be intact, with no curvature deformity or abnormal swelling and no bone defects. In contrast, significant differences were observed in mice infected with MRSA. There were several notable features in the 2D and 3D images, including abnormal morphology in the proximal tibia, a curved tibia, and greater enlargement than that of the control group; these observations revealed a considerable progression of PTO. As opposed to the control group, all three treatment groups, especially the HBPL‐gel group, inhibited the progression of bacterial invasion. The micro‐CT images showed evidence for this inhibition. Subsequently, we analyzed the structural and density indices of the entire tibias (Figure 8d). As presented, a tendency toward higher bone mineral density (BMD) was observed in all three treatment groups. Thus, we concluded that the treatment helped increase the total mineral content and mineralization of bone tissue. Additionally, the results indicated that trabeculae thickness (Tb. Th) in the treated groups was higher. From a structural point of view, HBPL possesses both the units of ε‐PL and PLL. The ε‐PL has been used as a preservatives and antibacterial agents,^[^ ^45^ ^]^ whereas the PLL has been used to promote cell attachment, proliferation and differentiation,^[^ ^46^ ^]^ which might also play an important role in the osteogenesis. Extensive results have shown that antibacterial hydrogel treatment can, in some cases, hinder bacterial invasion of bone marrow, encourage the synthesis and repair of bone tissue, and improve bone mass.

### 2.8 Biotoxicity and Safety

**Figure 9:**
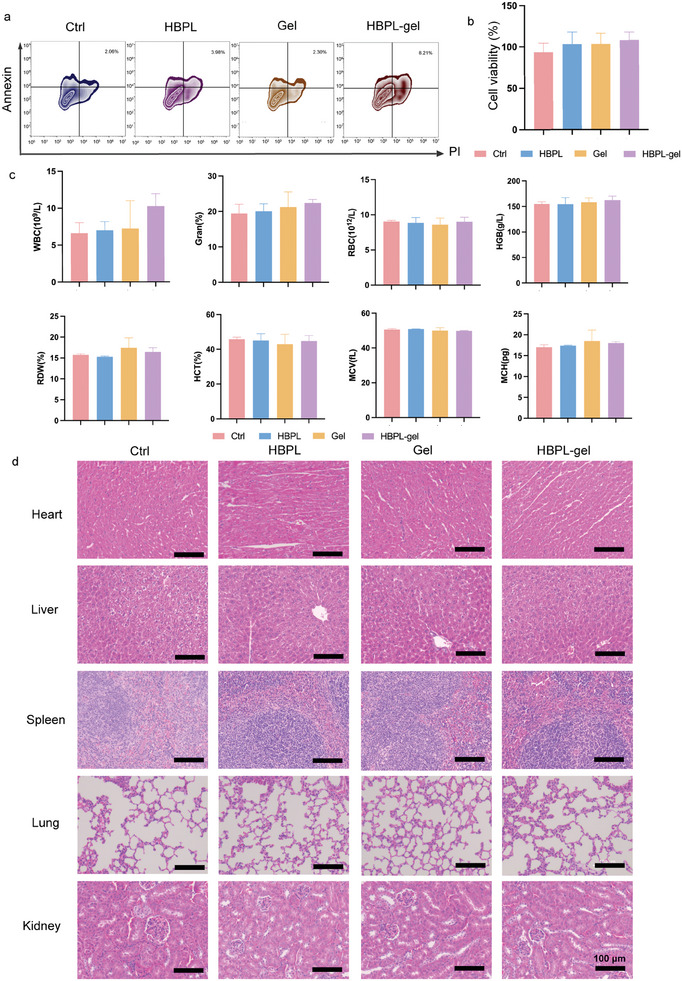
Preliminary toxicity and security of hydrogel in vitro and in vivo. a) Flow cytometry of BMSCs stained with Annexin and PI under different treatments. b) Cell viabilities of BMSCs co‐incubated with different treatments. c) Blood routine (WBC, white blood cell; Gran, polymorphonuclear neutrophil; RBC, red blood cell; HGB, hemoglobin; RDW, red blood cell distribution width; HCT, hematocrit; MCV, mean cell volume; MCH, mean corpuscular hemoglobin) of healthy mice under different treatments. d) H&E staining of heart, liver, spleen, lung, and kidney of healthy mice under different treatments. Data are presented as mean ± SD. (*n* = 3–5 per group).

To assess the toxicity of antimicrobial hydrogels in vitro, we incubated each group of hydrogel infusions with bone mesenchymal stem cells (BMSCs) and observed the morphology of BMSCs under a microscope. We found that neither the hydrogel nor the QS inhibitor affected the normal growth of BMSCs. In addition, we performed Annexin/PI staining of each group of cells after incubation and analyzed apoptosis by flow cytometry, which illustrated that the apoptosis level of each group was within the normal range (**Figure** **9**a). Subsequently, we verified the toxic effects of hydrogels on BMSCs by Cell counting kit 8 (CCK8), and the results showed that the survival rate of BMSCs was close to 100% after incubation with each group of hydrogels for 12 h (Figure 9b). This further confirmed the safety of the antimicrobial hydrogel on BMSCs in vitro.

Moreover, we estimated the toxicity of the antimicrobial hydrogel in vivo. On day 14, no abnormalities, such as significant rupture, redness, pustules, or erythema, were found at the wound site in all groups. In addition, there was no significant change in the routine blood indices (Figure 9c) in the treated or untreated groups. The H&E staining results obtained for important organs (heart, liver, spleen, lung and kidney) showed no significant effect on their histological structures (Figure 9d), and these results indicate that the antibacterial hydrogel shows great biosafety in vivo.

Together, the present findings confirm that this antibacterial hydrogel exhibits a high safety profile, which provides a good basis for future translation into clinical studies.

## 3 Conclusion

In summary, we provide a prospective nonantibiotic dressing strategy for the treatment of PTO. The HBPL contained in the dressing could effectively kill bacteria in the repair location and prevent the formation of PTO. The antibacterial hydrogel dressing could promote bone repair by integrating debridement, disinfection, repair promotion and dressing. Thus, the hydrogel can effectively prevent the occurrence and sequelae of PTO caused by multidrug‐resistant bacterial infections, such as MRSA, through timely treatment in some complex field environments.

## 4 Experimental Section

### Materials

PVA was purchased from Innochem, China; Hydroxyl Radical Scavenging Ability Colorimetric Test Kit was purchased from Elabscience, USA; Inhibition and Production of Superoxide Anion Radical Colorimetric Test Kit was purchased from Elabscience, USA; DPPH radical scavenging ability test kit was purchased from Solarbio, China. Live/dead BacLight Bacterial Viability Kit (L7012) was purchased from ThermoFisher Scientific, USA. Tryptophan soy broth (TSB) and TSA medium were purchased from Solarbio, China. CCK‐8 was purchased from Dalian Meilunbio, China. MRSA, USA300 ATCC‐BAA‐1556 strain was obtained from the American Type Culture Collection. Luci‐MRSA was constructed by Forhigh Biotech, China. The reverse transcription kit was purchased from Biosharp, China. qPCR SYBR Green Master Mix was purchased from Yeasen, China. Reactive Oxygen Assay Kit was purchased from Yeasen, China. Male C57BL/6 mice, 6–8 weeks, were purchased from SLAC Laboratory Animals, Shanghai, China.

### Synthesis of Gel

0.5 g 4‐ (Bromomethyl)phenylboronic acid and 0.1 g N, N, N′, N′‐Tetramethyl‐1,3‐propanediamine were added into a 25 mL round bottom flask and 10 mL anhydrous N, N‐dimethylformamide was added. Next, the temperature of the reaction system was adjusted to 60 °C overnight. Subsequently, the mixture was cooled to room temperature, and 100 mL tetrahydrofuran was added to wait for the white solid to precipitate and dry. Then, the same volume of aqueous PVA solution was mixed with TSPBA to obtain a hydrogel.

### Characterization of Gel

The sample was dried, mixed with KBr, fully ground, pressed into a transparent sheet on the hydraulic press, and then placed in the infrared spectrometer for testing. The tensile and compressive properties of the hydrogels were tested by mechanical property testing (ZwickRoell, Germany). Rheological properties were tested by a rheometer (Haake, Germany). To investigate the release rate, samples were immersed in different solutions and gently shaken at 37 °C. After certain times, 100 µL of supernatant was collected for polypeptide quantification by measuring the absorbance at 215 nm.

### Antioxidant Ability Detection

The scavenging abilities of ·OH, ·O_2_^−^, DPPH and H_2_O_2_ were detected by the Hydroxyl Free Radical Scavenging Capacity Assay Kit (Elabscience, USA), Inhibition and Production of Superoxide Anionic Colorimetric Assay Kit (Elabscience, USA), DPPH Free Radical Scavenging Capacity Assay Kit (Solarbio, China), and Hydrogen Peroxide Colorimetric Assay Kit (Elabscience, USA) respectively, according to the manufacturer's instructions. Briefly, samples were mixed with working solution and incubated at 37 °C. The absorbance was detected by a microplate reader (Infinite M200 Pro, Tecan, Switzerland) at set time intervals.

### ROS Clearance Efficiency Detection

RAW264.7 murine macrophage cells were cultured in Dulbecco's modified eagle medium containing 10% fetal bovine serum, 100 U mL^−1^ penicillin and 100 µg mL^−1^ streptomycin at 37 °C in a 5% CO_2_ atmosphere. The cells were seeded in a 12‐well plate. After cells attachment, the medium was replaced by serum‐free‐medium with 100 µM H_2_O_2_. Then different hydrogels samples were injected. Cells were stained by DCFH‐DA (Yeasen, China), and imaged with an SP8 Lighting confocal microscope (Leica, Germany).

### Bacteria Counting

MRSA were cultured in TSB medium. The bacterial solution was centrifuged (4000 rpm, 10 min) and resuspended in PBS. The absorbance of the bacterial suspension at 600 nm was measured by a microplate reader (Infinite M200 Pro, Tecan, Switzerland). Then, the bacterial suspensions were diluted and coated on TSA plates at 37 °C for 12 h. Photos were obtained, and Image J was used for colony counting.

### Bacteria Live/Dead Detection

MRSA suspensions were incubated with PBS, hydrogel, HBPL and HBPL‐gel for 24 h before live/dead detection. Pretreated bacterial cells were incubated with SYTO 9 and PI solution at 37 °C for 15 min (ThermoFisher Scientific, USA). Then, live and dead cells were imaged with an SP8 Lighting confocal microscope (Leica, Germany).

### Biofilm Formation Detection

MRSA suspensions were cultured at 37 °C for 72 h to form biofilms. Then, the biofilms were treated with PBS, hydrogel, HBPL and HBPL‐gel for 3 h. After treatment, the biofilms were washed with PBS and stained with SYTO 9 and PI. Finally, 3D images were taken with an SP8 Lighting confocal microscope (Leica, Germany).

### TEM

MRSA was incubated with PBS, hydrogel, HBPL and HBPL‐gel for 24 h. Then, the bacteria were fixed with 2.5% glutaraldehyde, washed with PBS, and dehydrated with ethanol and acetone in turn. The bacterial morphology was observed by TEM (Tecnai G2 spirit, USA).

### Building the Mouse PTO Models

All animal experiments were conducted by the institutional regulations for Animal Care and Use Committee of Second Affiliated Hospital, Zhejiang University School of Medicine. Six‐ to eight‐week‐old male C57BL/6 mice (25–30 g) were used to establish the PTO model. The whole process was performed in a sterile table. After anesthesia, the animals were depilated and disinfected, and a skin incision was created in the right leg. Then, the tibial plateau of the right leg was exposed, and a hole was created to form a fracture trauma model. The fracture site was injected with MRSA solution (1 × 10^7^ CFU). The wound was glued with tissue adhesive. Subsequently, the mice were randomly distributed into the following groups: Ctrl group, HBPL group, Gel group and HBPL‐gel group. The wounds were covered with 3m Tegaderm film. Finally, the treatments were repeated every 2 days. Mice were euthanized at 7 days and 14 days post infection. Tibiae and the surrounding soft tissues were surgically removed, photographed, and prepared for subsequent experiments.

### qPCR

The primers used in this study are listed in Table S1 (Supporting Information). Tissues were fully lysed with TRIzol. RNA was reverse transcribed into complementary DNA using a reverse transcription kit. qPCR was conducted in a 20 µL system, including 2 µL of cDNA, 0.4 µL of forward and reverse primers, 10 µL of SYBR, and 7.2 µL of double distilled water. Relative expression was normalized to the *Gapdh* level.

### In Vivo Fluorescence Imaging

For in vivo fluorescence imaging, Luci‐MRSA was used for to establish the PTO model. After the corresponding treatments mentioned above were performed, each mouse was intraperitoneally injected with luciferin potassium. The chemiluminescent signals were measured by an IVIS. The fluorescence intensity of the ROI was analyzed by IVIS.

### Histological Staining

Tissues were collected and fixed with 4% paraformaldehyde and embedded in paraffin. Here, tibiae were decalcified in 10% EDTA in addition. H&E staining, Masson staining, Gram staining, immunofluorescence staining and immunohistochemical staining were carried out using standard techniques. Images were taken with an upright microscope (Leica, Germany).

### Histological Analyses

Based on Smeltzer's scores,^[^ ^33^ ^]^ histological results were scored from 0 to 4 in intraosseous acute inflammation, intraosseous chronic inflammation, periosteal inflammation and bone necrosis. Higher scores indicate more severe inflammation. Images and slides were blinded for analysis and counted by ImageJ. All the scores were completed independently by two professionals who were not involved in the animal experiments.

### Micro‐CT

Mouse tibiae were isolated at certain times, fixed with paraformaldehyde, and scanned by high‐resolution micro‐CT (Milabs, Netherlands). 3D reconstruction of the images was performed by MILabs Rec 10.16, and the data were analyzed by IMALYTICS Preclinical Version 2.1.8.9. Volumes were segmented using a global threshold of 1600. The 360° scan angle, 20 µm voxel size resolution and 50 KVp were used for scans. Images were blinded for analysis, and all the scores were completed independently by two professionals.

### Toxicity and Safety Studies

For the in vitro assay, BMSCs were seeded in 96‐well plates. After the indicated treatment for 24 h, cell viability was measured by CCK‐8 (Meilun Biotechnology, China) and Annexin/PI double staining kits (Yeasen, China). For the in vivo assay, normal mice were used. After anesthesia, depilation and disinfection were performed, a fracture wound was created in the right leg. Then, the wound was injected with PBS, hydrogel, HBPL and HBPL‐gel. On day 14, the mice were euthanized. The major organs were isolated (heart, lung, liver, spleen, and kidney), fixed with 10% paraformaldehyde, dehydrated, paraffin‐embedded, sectioned and stained with H&E for histological examination. Routine blood tests were performed.

### Statistical Analysis

The data were presented as the mean ± SD and analyzed by GraphPad Prism 9. All experiments were performed at least three times to ensure reproducibility. Significant differences were analyzed through one‐way analysis of variance (ANOVA) by Tukey's HSD. A *p* value of < 0.05 was considered statistically significant .

## Conflict of Interest

The authors declare no conflict of interest.

## Author Contributions

W.Z., H.L., and W.Z. contributed equally to this work. W.Z. and F.X. participated in conception and design of this study; W.Z., H.L., and W.Z designed, analyzed, and performed all the experiments; J.H., Y.Z., H.H., L.S., and J.X. provided the experimental technical support. W.Z. wrote the manuscript. L.S. and F.X. supervised the research and revised the paper. All authors contributed to manuscript revision and final approval of the manuscript. The authors thank Chenyu Yang in the Center of Cryo‐Electron Microscopy, Zhejiang University for their technical assistance on transmission electron microscope. The authors thank Xiaoli Hong from the laser scanning confocal microscope, Zhejiang University School of Medicine for their technical support.

## Supporting information

Supporting Information

Supplementary Video 1

Supplementary Video 2

## Data Availability

The data that support the findings of this study are available on request from the corresponding author. The data are not publicly available due to privacy or ethical restrictions.
