# Supplementary material for: Inflammatory Microenvironment‐Responsive Hydrogels Enclosed with Quorum Sensing Inhibitor for Treating Post‐Traumatic Osteomyelitis
Source: Adv Sci (Weinh). 2024 Mar 14;11(20):2307969. doi: 10.1002/advs.202307969 (PMC11132068; doi:10.1002/advs.202307969)
Supplement: Supplementary file 1 — Supporting Information [file ADVS-11-2307969-s001.pdf]

ADVANCED SCIENCE
Open Access

Supporting Information

for *Adv. Sci.*, DOI 10.1002/advs.202307969

Inflammatory Microenvironment-Responsive Hydrogels Enclosed with Quorum Sensing Inhibitor for Treating Post-Traumatic Osteomyelitis

*Wenting Zhang, Huidan Lu, Wanying Zhang, Jiahao Hu, Yifei Zeng, Huiqun Hu, Liyun Shi, Jingyan Xia and Feng Xu**

Supplementary Information

Inflammatory Microenvironment-Responsive Hydrogels Enclosed With Quorum Sensing Inhibitor for Treating Post-Traumatic Osteomyelitis

Wenting Zhang [1, 2, 3], Huidan Lu [1, 2, 3], Wanying Zhang [1, 2, 3], Jiahao Hu [4], Yifei Zeng [1, 2, 3], Huiqun Hu [1, 2, 3], Liyun Shi [5], Jingyan Xia [6], Feng Xu [1, 2, 3, *]

[1]Department of Infectious Diseases, the Second Affiliated Hospital, Zhejiang University School of Medicine, Hangzhou, Zhejiang, 310009, China;

[2]Key Laboratory of Multiple Organ Failure (Zhejiang University), Ministry of Education, Hangzhou, 310053, China;

[3]Research Center for Life Science and Human Health, Binjiang Institute of Zhejiang University, Hangzhou, 310053, China;

[4]Department of General Surgery, Sir Run-Run Shaw Hospital, Zhejiang University School of Medicine, Hangzhou, Zhejiang, 310016, China;

[5]Institute of Translational Medicine, Zhejiang Shuren University, Hangzhou, Zhejiang 310015, China.

[6]Department of Radiation Therapy, Second Affiliated Hospital, Zhejiang University School of Medicine, Hangzhou, Zhejiang, 310009, China;

***Correspondence:**

F.X. (Email: xufeng99@zju.edu.cn)

Supporting table

Table S1. Primers used for qPCR in this study.

Primers	Sequence (5′ → 3′)	Primers	Sequence (5′ → 3′)
Il-1β-F	GCAACTGTTCCTGAACTCAACT	***Il-1β-R***	ATCTTTTGGGGTCCGTCAACT
Il-6-F	TAGTCCTTCCTACCCCAATTTCC	***Il-6-R***	TTGGTCCTTAGCCACTCCTTC
Tnf-F	CCCTCACACTCAGATCATCTTCT	***Tnf-R***	GCTACGACGTGGGCTACAG

Supporting figures

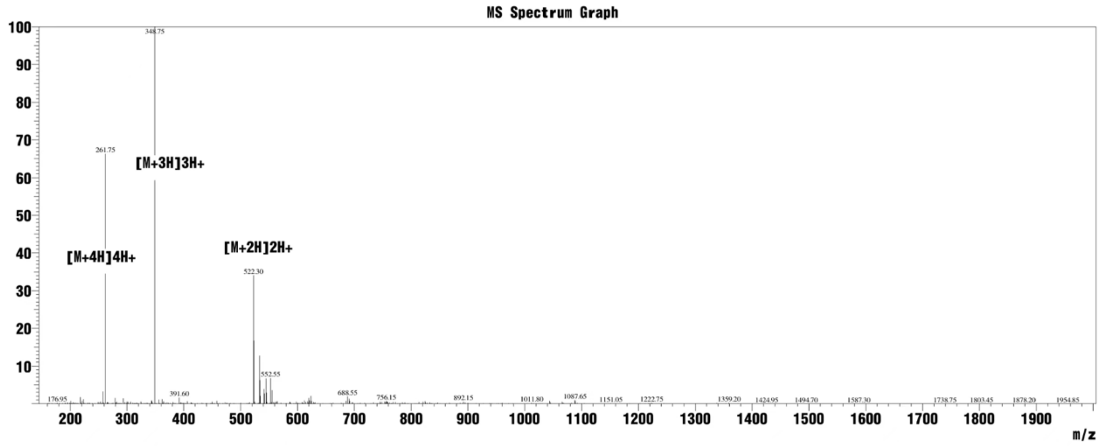

Fig. S1 Mass Spectrometry of HBPL.

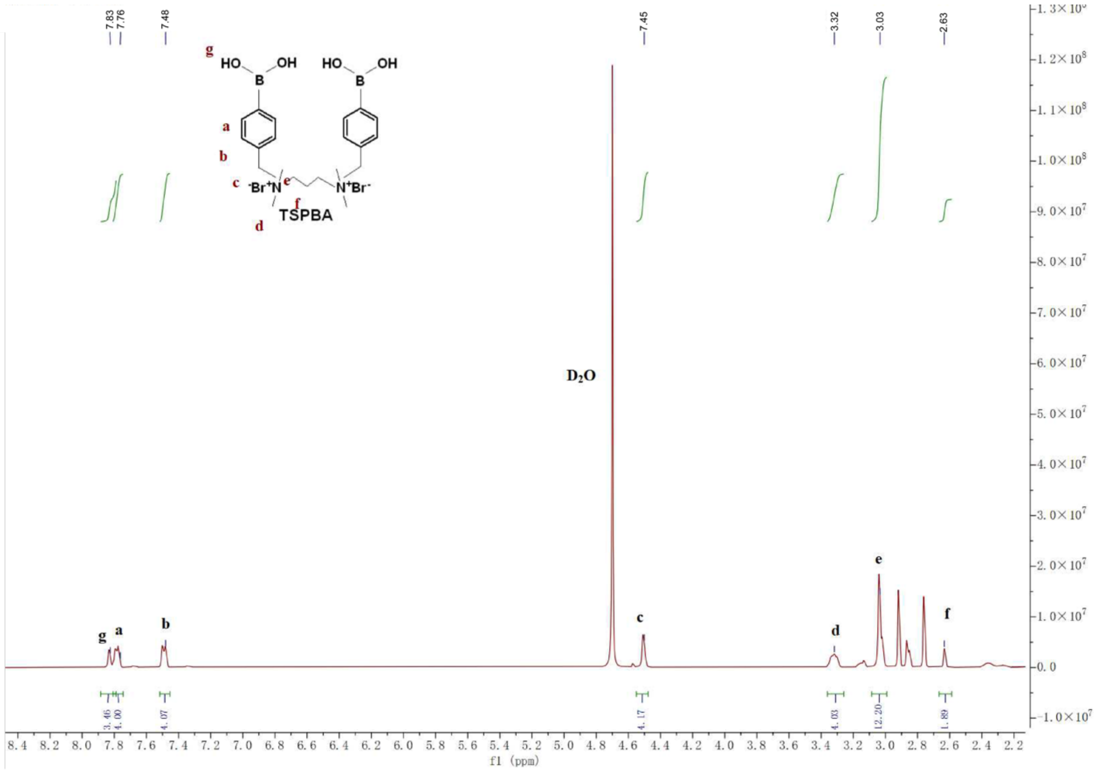

Fig. S2 Nuclear Magnetic Resonance of TSPBA.

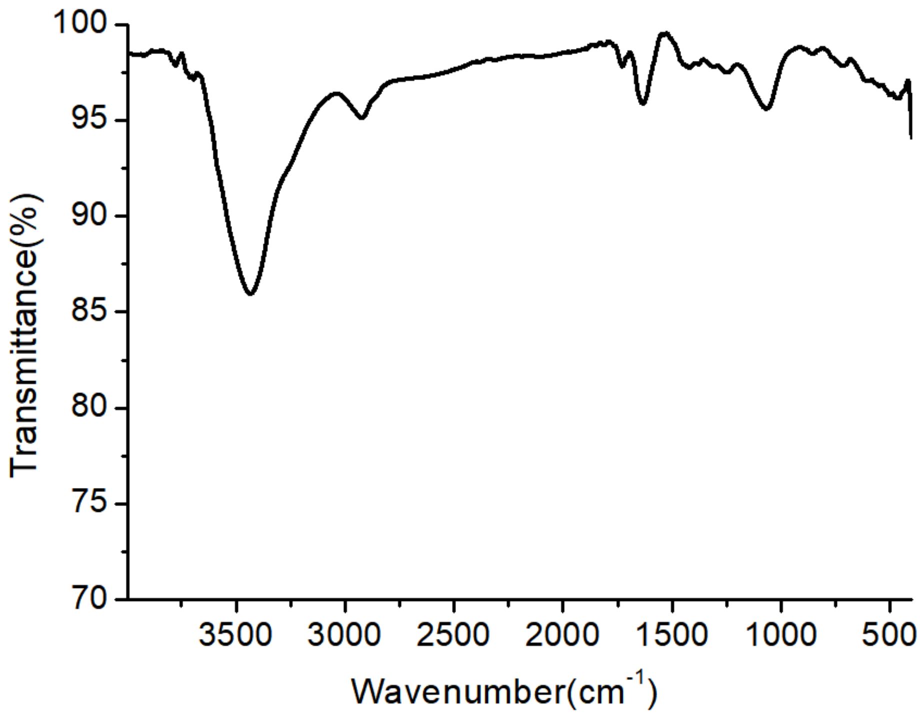

Fig. S3 FTIR spectrum of TSPBA-PVA hydrogels.

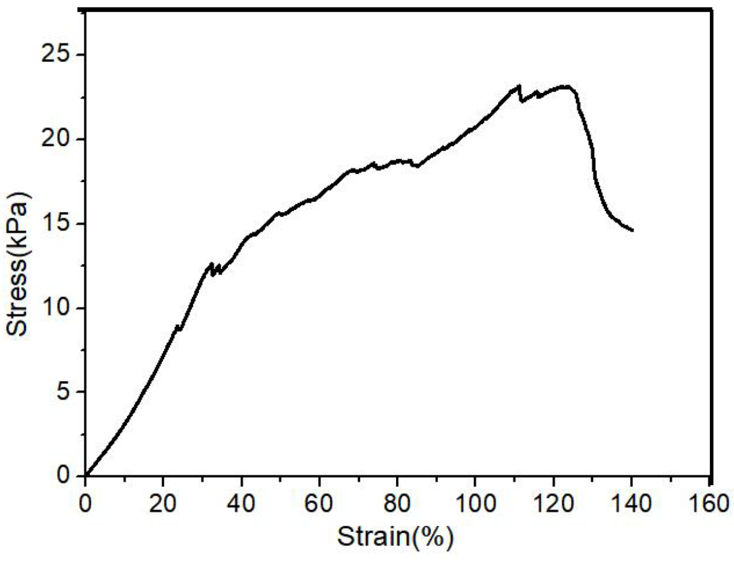

Fig. S4 Shear thinning properties of TSPBA-PVA hydrogels.

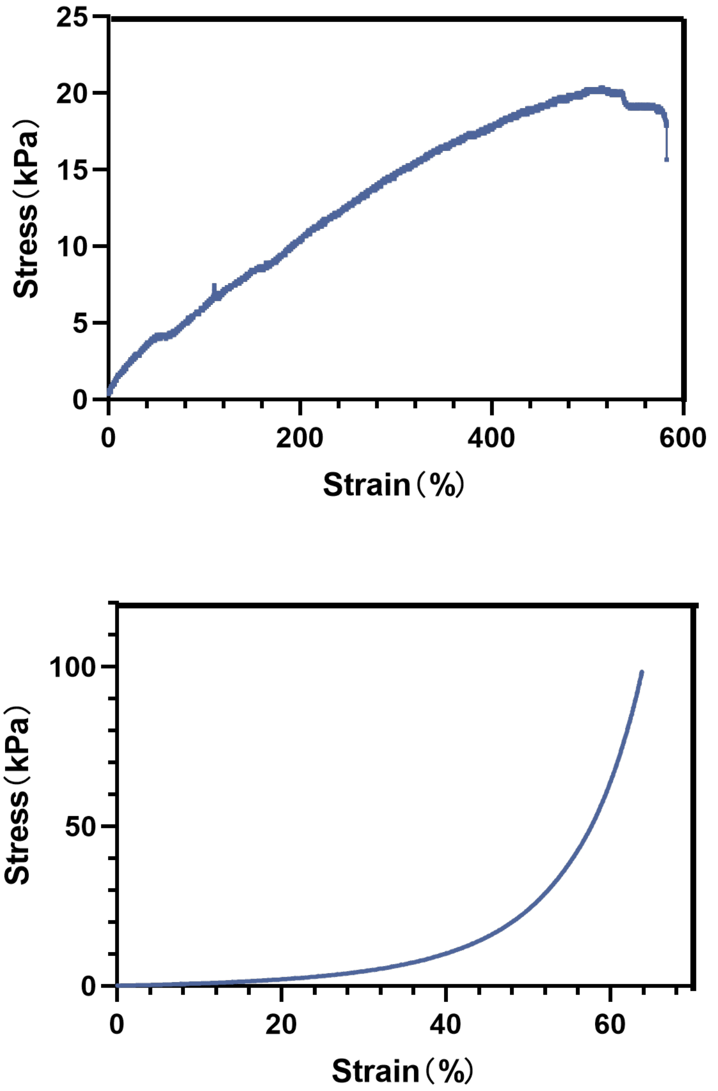

Fig. S5 The mechanical properties of TSPBA-PVA hydrogels including tensile and compressive properties.

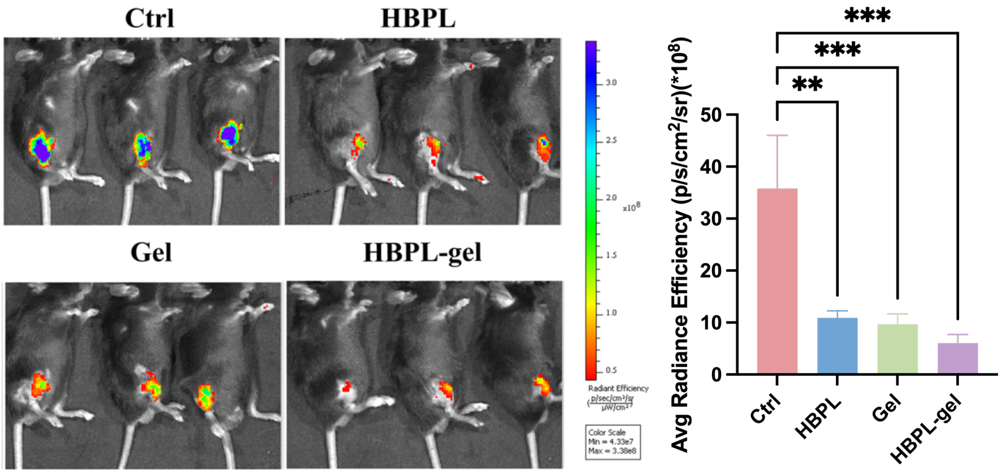

Fig. S6 Representative images of in vivo imaging system and fluorescence quantitative analysis after injection of ROS Brite™ 700. Data are presented as mean ± SD. (n = 3-5 per group, ** $p<0.01$, *** $p<0.001$)
